# Establishment of a risk prediction model for residual low back pain in thoracolumbar osteoporotic vertebral compression fractures after percutaneous kyphoplasty

**DOI:** 10.1186/s13018-024-04528-y

**Published:** 2024-01-06

**Authors:** Weiqiao Tu, Yanping Niu, Peng Su, Di Liu, Fanguo Lin, Yongming Sun

**Affiliations:** Department of Orthopedics, Second Affiliated Hospital of Soochow University, No. 1055, Sanxiang Road, Suzhou, Jiangsu People’s Republic of China

**Keywords:** Osteoporotic vertebral fractures, Residual low back pain, Potential risk factors, Prediction model, Percutaneous kyphoplasty

## Abstract

**Objective:**

This study aims to identify potential independent risk factors for residual low back pain (LBP) in patients with thoracolumbar osteoporotic vertebral compression fractures (OVCFs) following percutaneous kyphoplasty (PKP) treatment. Additionally, we aim to develop a nomogram that can accurately predict the occurrence of residual LBP.

**Methods:**

We conducted a retrospective review of the medical records of thoracolumbar OVCFs patients who underwent PKP treatment at our hospital between July 2021 and December 2022. Residual LBP was defined as the presence of moderate or greater pain (VAS score ≥ 4) in the low back one day after surgery, and patients were divided into two groups: the LBP group and the non-LBP group. These patients were then randomly allocated to either a training or a validation set in the ratio of 7:3. To identify potential risk factors for residual LBP, we employed lasso regression for multivariate analysis, and from this, we constructed a nomogram. Subsequently, the predictive accuracy and practical clinical application of the nomogram were evaluated through a receiver operating characteristic (ROC) curve, a calibration curve, and a decision curve analysis (DCA).

**Results:**

Our predictive model revealed that five variables—posterior fascial oedema, intravertebral vacuum cleft, time from fracture to surgery, sarcopenia, and interspinous ligament degeneration—were correlated with the presence of residual LBP. In the training set, the area under the ROC was 0.844 (95% CI 0.772–0.917), and in the validation set, it was 0.842 (95% CI 0.744–0.940), indicating that the model demonstrated strong discriminative performance. Furthermore, the predictions closely matched actual observations in both the training and validation sets. The decision curve analysis (DCA) curve suggested that the model provides a substantial net clinical benefit.

**Conclusions:**

We have created a novel numerical model capable of accurately predicting the potential risk factors associated with the occurrence of residual LBP following PKP in thoracolumbar OVCFs patients. This model serves as a valuable tool for guiding specific clinical decisions for patients with OVCFs.

## Introduction

Osteoporosis is a systemic metabolic bone disorder characterized by decreased bone mass and the destruction of trabecular bone structure, which often leads to fragility fractures [1]. Osteoporotic vertebral compression fractures (OVCFs), a prevalent complication of osteoporosis-related fragility fractures [2, 3], are frequently associated with chronic and persistent low back pain (LBP), progressive spinal deformity, and other issues, significantly impacting the patients' daily quality of life. In clinical practice, OVCFs can be managed through both conservative and surgical approaches. While conservative treatment of OVCFs has shown relatively high mortality rates, PKP has emerged as an effective treatment [4, 5], providing substantial relief from back pain, spinal stability restoration, early patient mobilization, and prevention of complications such as hypostatic pneumonia and pressure ulcers [6, 7]. However, our clinical observations have revealed that some patients continue to experience residual LBP after undergoing PKP. Previous research studies have reported various potential risk factors associated with the persistence of LBP following PKP [8], including factors such as non-healing bone cement interfaces, thoracolumbar fascia injury (TLFI), cement volume and distribution, and IVC. Some studies have developed risk prediction models for residual LBP after PKP. However, previous models had a limited set of potential independent risk factors. In our study, we conducted a comprehensive analysis of various baseline factors related to postoperative residual LBP in patients with OVCFs. We aimed to develop a predictive model that can be utilized by physicians to inform clinical decision-making for OVCFs patients.

## Methods

### Patients

**Fig. 1:**
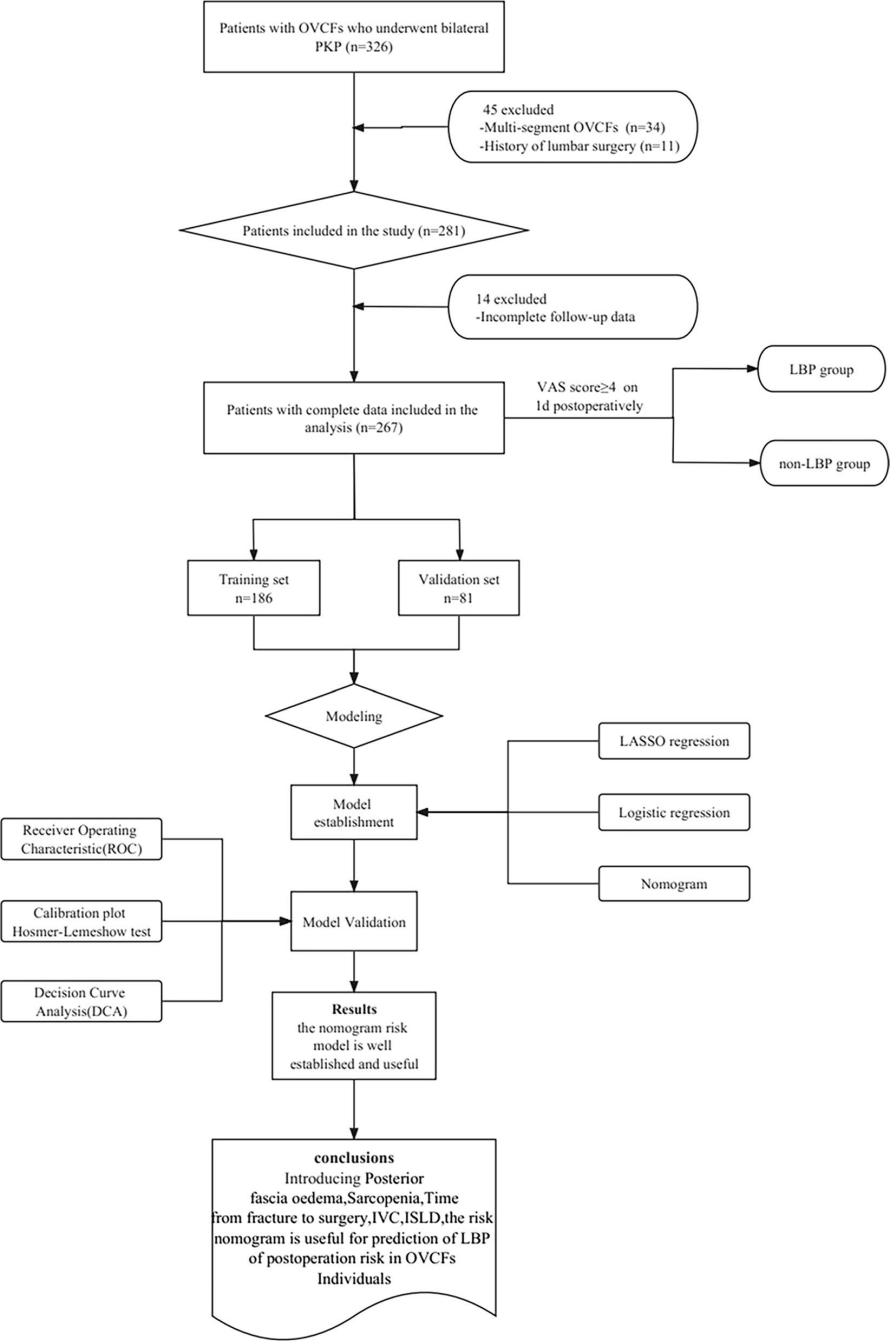
Flowchart of study design

Clinical data of patients with single-segment thoracolumbar OVCFs who underwent a bilateral pedicle approach PKP from July 2021 to December 2022 in our hospital were retrospectively analyzed. The flowchart of our study design is shown in Fig. 1. The specific criteria for inclusion and exclusion were as outlined below. Inclusion criteria: (1) Low back pain due to low-energy injury (e.g., fall, sprain, or lifting a heavy object); (2) X-ray showing a single-segment thoracolumbar vertebral fracture (T10-L2) and prominent high signal intensity on magnetic resonance imaging (MRI) fat-suppressed images of the fractured vertebral body; (3) Dual-energy X-ray absorptiometry (DXA) T-scores of ≤ − 2.5 in the spine/hip; and (4) No symptoms of nerve root and/or spinal cord compression. And exclusion criteria were as follows: Visual analogue scale (VAS) pain score < 5. (2) OVCFs caused by tumor, infection, or tuberculosis, etc. (3) Fracture of the vertebral column in the posterior region, with bone fragments displaced into the spinal canal; and (4) Incomplete follow-up data.

### Surgical technique

After the patient was successfully placed under general anesthesia, the patient was positioned in a prone posture. Using the C-arm machine for fluoroscopy, the bilateral arches of the fractured vertebrae were precisely located, and the skin incision sites were marked with a marking pen. Following standard iodine disinfection procedures and the placement of a sterile towel, an incision of approximately 0.5 cm was made at each marked site. Subsequently, a guide needle was carefully inserted into the appropriate position along the outer edge of the vertebral pedicle, allowing the insertion of an expansion cannula to create a working channel. A balloon filled with contrast agent was then positioned to provide support for the vertebral body. Upon reaching the desired height for the vertebral body, the expansion process was halted, and the balloon was carefully removed. At this stage, the bone cement (Mendec, Italy) was introduced slowly into the fractured vertebra. The injection was ceased once the cement had sufficiently diffused to the posterior wall of the vertebral body. Following the solidification of the bone cement, the needle used for the procedure was removed, and the skin incision was covered with sterile gauze, marking the conclusion of the operation. Additionally, lumbar protection through the use of a lumbar brace was routinely employed for one month post-surgery.

### Postoperative management

After the surgery, all patients received postoperative anti-osteoporotic treatment [9, 10], which included oral calcium and vitamin D supplementation [11], as well as an annual intravenous infusion of zoledronic acid [12] (Aclasta, 100 ml/5 mg) for a period of three years. And all patients also received biochemical markers of bone turnover (BTMs) testing [13, 14], such as the bone alkaline phosphatase (bALP), procollagen type I N propeptide (PINP). Additionally, patients underwent routine orthopedic radiographic evaluations the day following the surgery to assess the distribution and diffusion of cement within the fractured vertebral body. Subsequent evaluations were conducted at one day, one month, and three months post-surgery to measure the mean severity of LBP and daily dysfunction. To evaluate pain severity and functional status in patients with OVCFs, VAS and Oswestry disability index (ODI) were employed. Residual LBP, as defined in this study, was characterized by a VAS score of ≥ 4 on the first day following surgery. Based on their VAS scores, patients were categorized into a LBP group and a non-LBP group.

### Outcome measures

Pre- and postoperative potential risk factors associated with the procedure were extracted from the medical record system, surgical records, radiology image management system, and questionnaires, including (1) demographic characteristics (age, BMI, gender, BMD) and preexisting medical conditions (history of diabetes, history of hypertension, etc.) (2) VAS, ODI, time from fracture to surgery, and preoperative radiological parameters (AVH, Cobb's angle, IVC, posterior fascial oedema, ISLD, and sarcopenia) (3) Postoperative radiographic parameters (cement volume, leakage, cement distribution, and changes in AVH and Cobb angle). All measurements were performed independently by three spine surgeons with 5 years of experience.

**Fig. 2:**
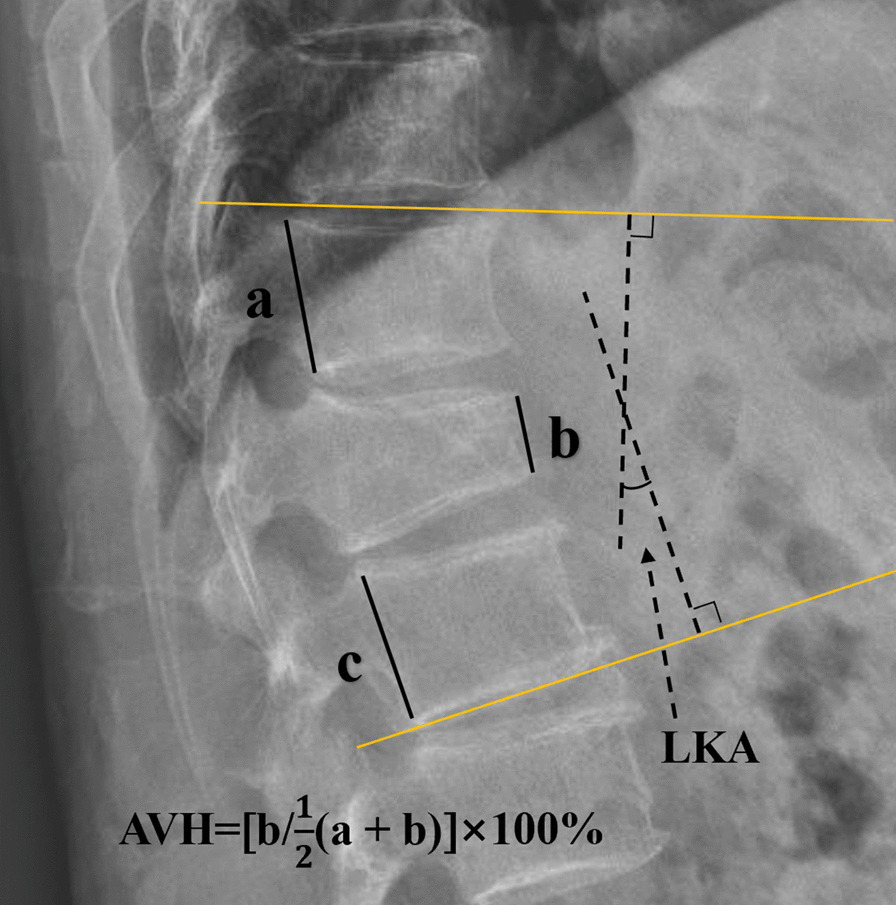
Radiographic evaluation of compressed vertebrae. **a** Posterior height of the upper vertebrae; **b** anterior height of the vertebrae; **c** posterior height of the lower vertebrae; LKA: local kyphotic Cobb’s angle

**Fig. 3:**
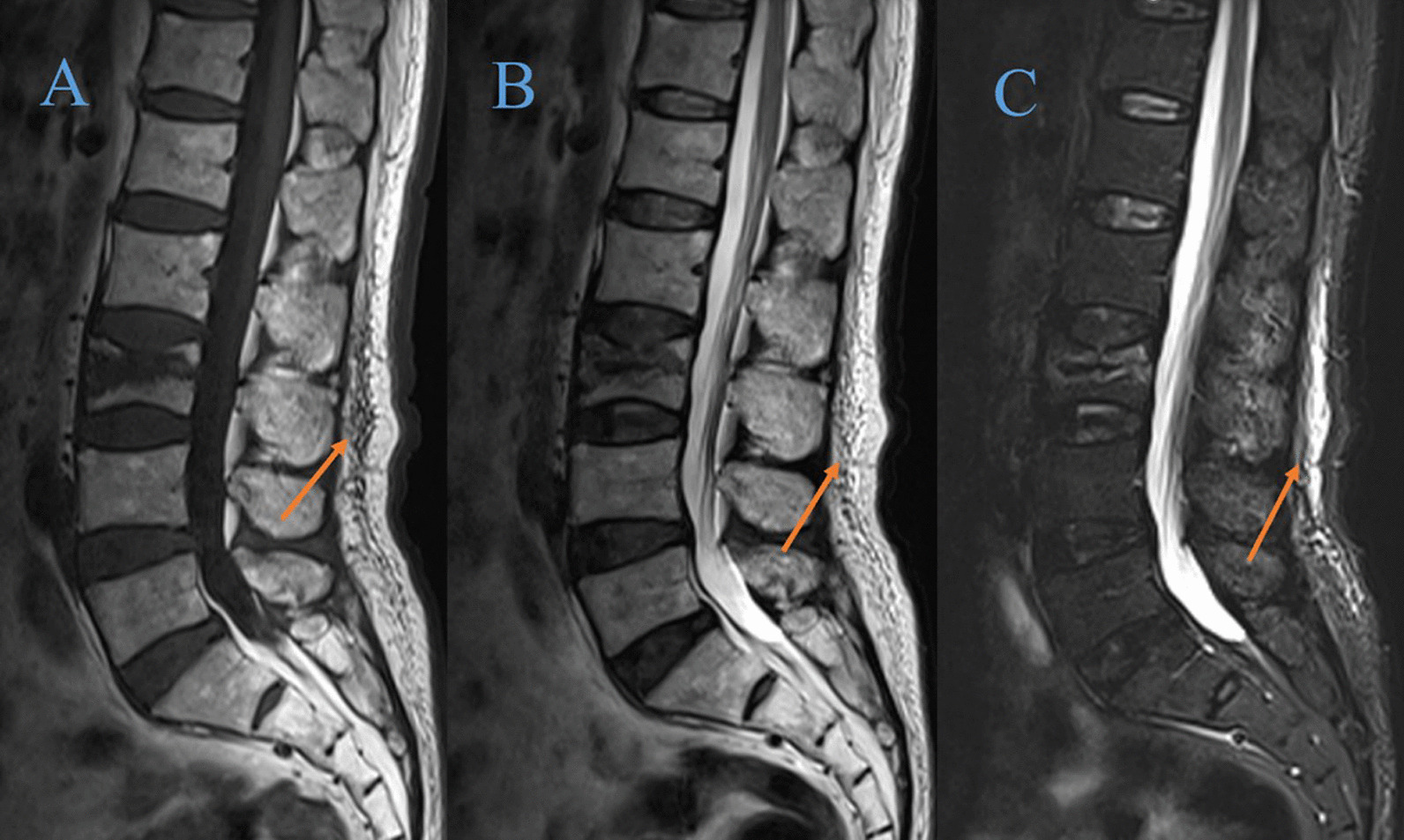
Posterior fascia oedema was visible on T1-WI (A, orange arrow), T2-WI (B, orange arrow) or STIR images (C, orange arrow) 0f MRI

**Fig. 4:**
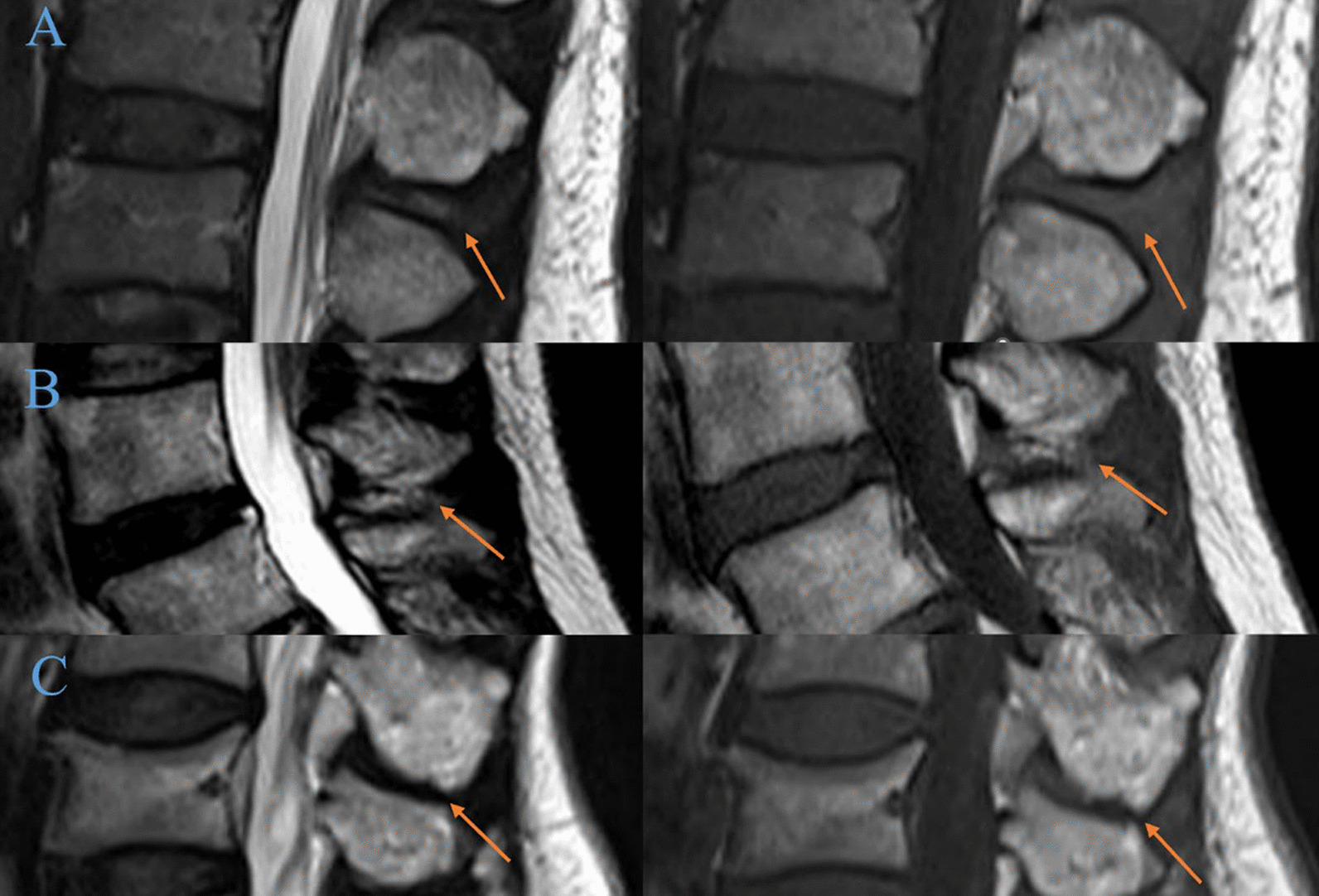
Example of each grade of interspinous ligament degeneration (orange arrow). **A** mild (high signal intensity on T1- and T2-WI). **B** moderate (low signal intensity on T1-WI and high signal intensity on T2-WI). **C** severe (low or iso-signal intensity on T1- and T2-WI with marked narrowing of the interspinous interval)

**Fig. 5:**
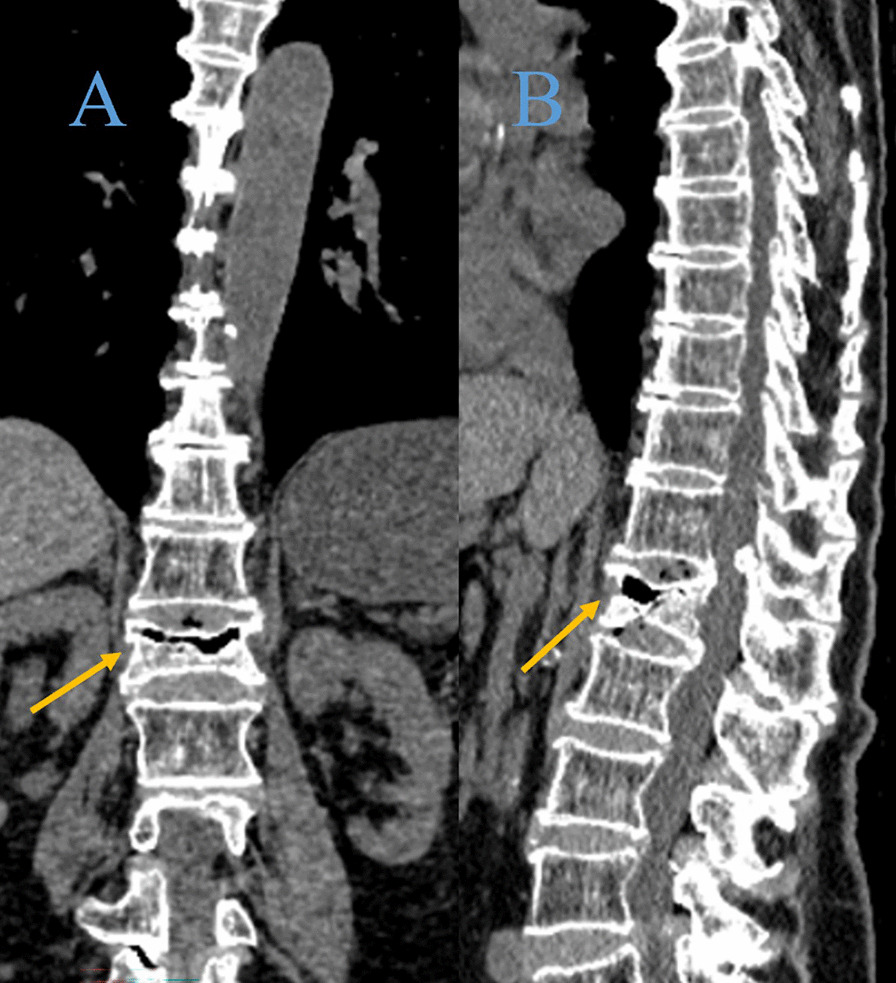
Intravertebral vacuum cleft was visible on coronal (**A**, yellow arrow) and sagittal (**B**, yellow arrow) views of CT

**Fig. 6:**
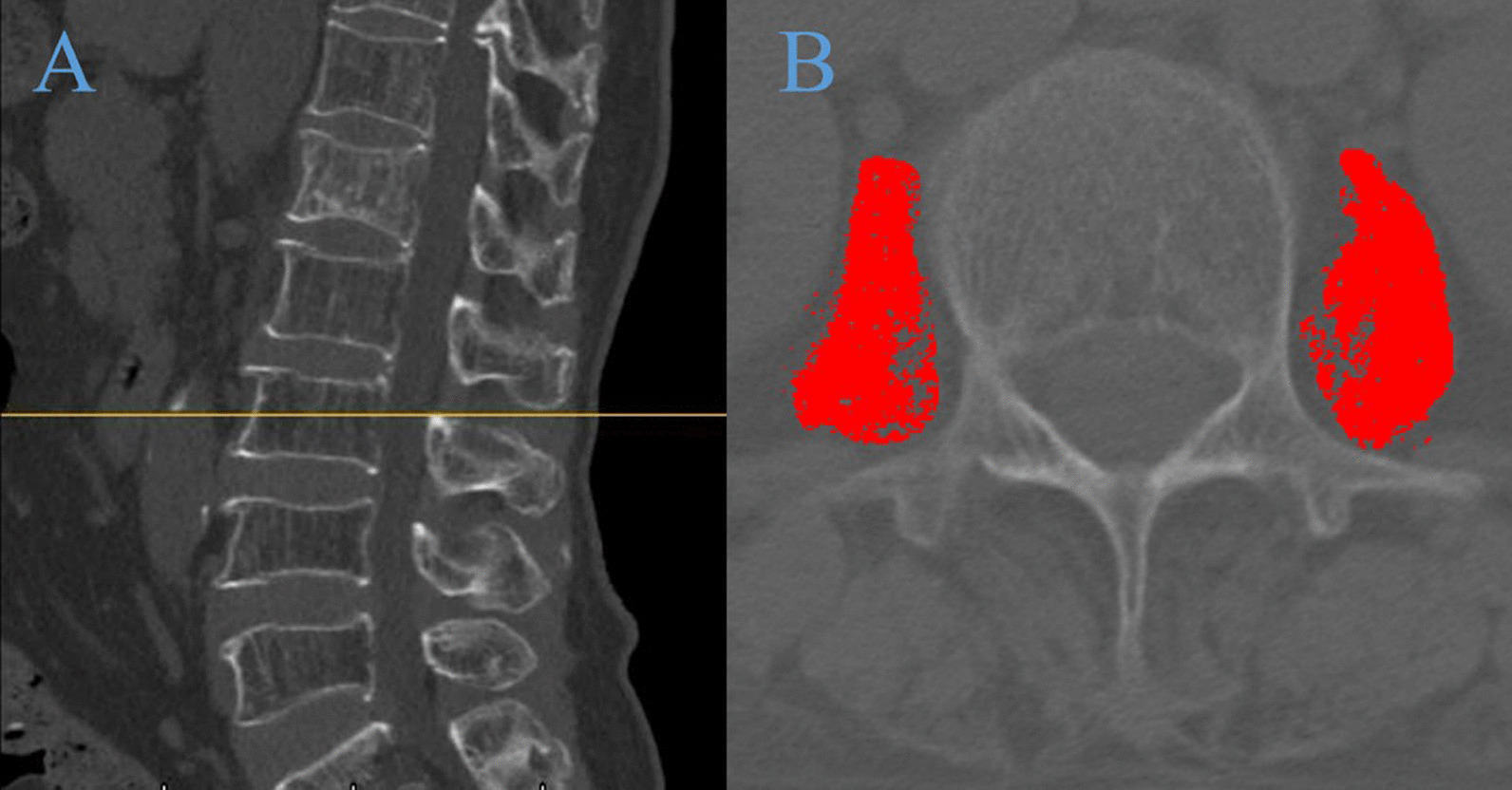
The TPA at level L3 was measured on CT by tracing the bilateral psoas major muscle outline. **A** sagittal views of Lumbar CT (L3, yellow line); **B** cross-sectional views of Lumbar CT (TPA, red fill)

The anterior vertebral height (AVH) and Cobb angle of the fractured vertebral body were measured prior to the surgical procedure and one day after surgery. The AVH of the fractured vertebra and the posterior border height of the adjacent normal vertebral body were measured on lateral radiographs using the method described by Teng et al. [15]. Due to the different presentation sizes of the radiographs, the vertebral body height was expressed as relative magnitude: (AVH of the fractured vertebral body /mean of the heights of adjacent posterior vertebral body) × 100% (Fig. 2). To assess the severity of the localized posterior convexity deformity, we used the local kyphotic Cobb’s angle (LKA) (Fig. 2). The definition of posterior fascial oedema was based on MRI (Fig. 3). The type of ISLD on MRI according to the classification of Keorochana et al. was shown in Fig. 4. Vertebral vacuum cleft (IVC) on CT was shown in Fig. 5. For the measurement of sarcopenia, the total psoas area (TPA) was measured based on the lumbar spine CT cross-sections. In brief, the CT image cross-section of the third lumbar vertebral transverse process was reviewed, and the outlines of bilateral psoas major were manually sketched (Fig. 6). The cross-sectional area was measured using the imaging system's software and averaged over three measurements. TPA was determined by dividing the cross-section area by the square of height, expressed as mm^2^/m^2^. A diagnosis of sarcopenia was made if the TPA was < 385 mm^2^/m^2^ in females or < 545 mm^2^/m^2^ in males.

### Statistical analysis

SPSS version 22.0 statistical software and R software (version 4.1.2) were used for data processing and generating relevant graphics. If continuous variables followed a normal distribution, they were expressed as *X̅* ± *S* and t-test was used for comparison between groups; otherwise, they were described as median and interquartile range (IQR) and used rank sum test to compare groups. Categorical variables were expressed as *n* (%), with group comparisons being performed through the Chi-squared (*X*^2^) test. The "glmnet" package was employed for the least absolute shrinkage and selection operator (LASSO) regression model, which aided in the selection of the most optimal predictive features [16]. Subsequently, we employed the "glm" package to create a numerical model through multivariate logistic regression analysis. Then, we made use of the "rms" package within the R software to construct the nomogram. Internal validation of the model was carried out through 500 iterations of bootstrap sampling using the "caret" package. ROC curves and calibration curves were plotted to evaluate the model's predictive performance and accuracy by using the "fbroc", "rms" packages. The "rmda" package was utilized to create a DCA plot, assessing the clinical utility of the risk prediction model. Differences were defined as statistically significant at *P* < 0.05.

## Results

In this study, we conducted postoperative follow-up for a total of 267 patients with OVCFs based on our inclusion and exclusion criteria. Among these patients, 46 (17.22%) experienced postoperative residual LBP, while the remaining 221 patients did not. As indicated in Table 1, the variances in preoperative VAS scores and Oswestry Disability Index (ODI) scores between the two groups did not demonstrate statistical significance (*P* > 0.05). In the postoperative assessments conducted at 1 day, 1 month, and 3 months, a significant difference in both VAS scores and ODI scores between the two groups was observed (*P* < 0.05).

**Table 1:** Summary of VAS and ODI after surgery between LBP group and non-LBP group

Follow-up	non-LBP group	LBP group	*P* value
Pre-VAS	7.00 [7.00, 8.00]	8.00 [7.00, 8.00]	0.059
1d-VAS	2.00 [2.00, 3.00]	4.00 [4.00, 5.00]	< 0.001
1 m-VAS	2.00 [1.00, 2.00]	2.00 [2.00, 3.00]	< 0.001
3 m-VAS	1.00 [1.00, 2.00]	2.00 [1.00, 2.00]	< 0.001
pre-ODI	72.0 [70.0, 74.0]	72.0 [70.0, 74.0]	0.746
1d-ODI	32.0 [30.0, 34.0]	34.0 [32.0, 36.0]	< 0.001
1 m-ODI	20.0 [18.0, 22.0]	20.0 [20.0, 23.5]	0.004
3 m-ODI	10.0 [10.0, 12.0]	12.0 [10.0, 14.0]	< 0.001

### Baseline patient characteristics in both the training and validation sets

We randomly divided the patients into a training set (186 patients) and a validation set (81 patients) in the ratio of 7:3. The fundamental characteristics of the OVCFs patients in both sets were shown in Table 2. Importantly, no statistically significant differences were observed in any of the variables between these sets (*P* > 0.05).

**Table 2:** Comparison of baseline characteristics between training set and validation set

Variables	Training set (*n* = 186)	Validation set (*n* = 81)	*P* value
Sex			0.608
Male	34 (18.3%)	12 (14.8%)	
Female	152 (81.7%)	69 (85.2%)	
Age(years)	71.5 [65.0,78.0]	71.0 [64.0,77.0]	0.697
BMI(kg/m2)	23.8 [21.8,25.3]	23.1 [21.2,25.2]	0.476
Bone mineral density (T score)	-2.60 [-3.10,-2.50]	-2.50 [-2.90,-2.50]	0.199
Dypertension history			0.796
Yes	87 (46.8%)	40 (49.4%)	
No	99 (53.2%)	41 (50.6%)	
Diabetes history			0.941
Yes	23 (12.4%)	11 (13.6%)	
No	163 (87.6%)	70 (86.4%)	
Time from fracture to surgery (4 weeks yes/no)			0.257
Yes	58 (31.2%)	19 (23.5%)	
No	128 (68.8%)	62 (76.5%)	
Posterior fascia oedema			0.492
Yes	33 (17.7%)	18 (22.2%)	
No	153 (82.3%)	63 (77.8%)	
IVC			1
Yes	23 (12.4%)	10 (12.3%)	
No	163 (87.6%)	71 (87.7%)	
Sarcopenia			0.999
Yes	43 (23.1%)	18 (22.2%)	
No	143 (76.9%)	63 (77.8%)	
Bone cement distribution			1
Yes	31 (16.7%)	13 (16.0%)	
No	155 (83.3%)	68 (84.0%)	
ISLD			0.922
Yes	44 (23.7%)	18 (22.2%)	
No	142 (76.3%)	63 (77.8%)	
Cement volume(ml)	6.00 [6.00, 8.88]	6.00 [6.00, 8.00]	0.825
Bone cement leakage			0.621
Yes	24 (12.9%)	8 (9.88%)	
No	162 (87.1%)	73 (90.1%)	
Pre-LKA (°)	17.0 [14.0, 23.0]	17.0 [14.0, 21.0]	0.687
Pro-LKA (°)	12.0 [9.00, 16.8]	12.0 [10.0, 15.0]	0.903
Pre-AVH (%)	59.0 [56.0, 61.0]	59.0 [57.0, 61.0]	0.625
Pro-AVH (%)	79.0 [77.2, 82.0]	79.0 [77.0, 81.0]	0.51

### Univariate analysis and variables screening using Lasso regression

**Fig. 7:**
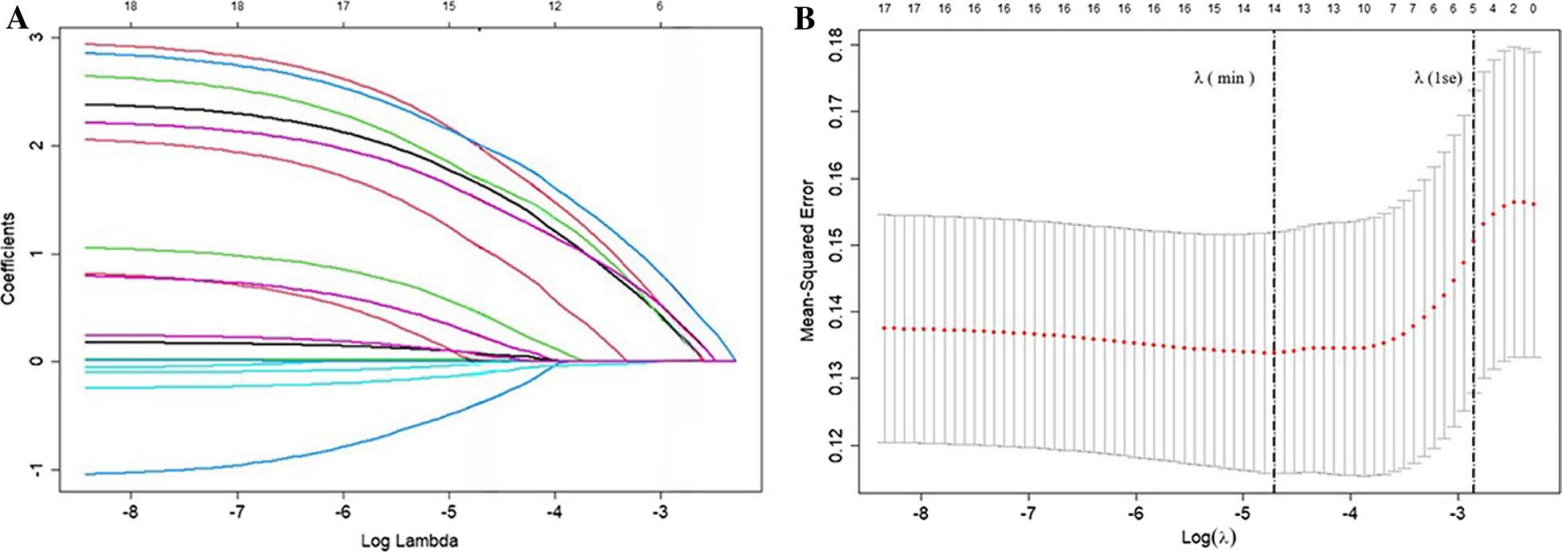
Variable selection by the LASSO binary logistic regression model. **A** LASSO coefficient profiles of the clinical features.  **B** The optimal penalization coefficient lambda was generated in the LASSO via tenfold cross-validation. We plotted the partial likelihood deviance (binomial deviance) curve versus log(lambda) and drew dotted vertical lines based on 1 standard error criteria. LASSO Least absolute shrinkage and selection operator

The outcomes of the univariate analysis for baseline demographic features, clinical and radiographic factors at baseline, as well as intraoperative factors, were presented in Table 3. When comparing the two groups, significant differences were observed in several variables: posterior fascial oedema (*P* = 0.022), IVC (*P* = 0.042), time from fracture to surgery (*P* = 0.016), sarcopenia (*P* = 0.001), and ISLD (*P* = 0.004). However, there were no statistically significant distinctions between the two groups for the remaining variables. To mitigate the influence of multicollinearity, confounding factors, and other issues among these variables, we conducted LASSO regression analysis. Ultimately, we selected five nonzero coefficient variables, as depicted in Fig. 7. These nonzero coefficient variables included posterior fascial oedema, IVC, time from fracture to surgery, sarcopenia, and ISLD.

**Table 3:** Results of Univariate analysis of variables associated with postoperative residual LBP in patients with OVCFs

Variables	β	OR	HR(95% CI)	*P* value
Sex (Male/Female)	0.557	1.745	1.745 (0.702–4.076)	0.21
Age (years)	− 0.02	0.98	0.98 (0.937–1.024)	0.38
BMI (kg/m^2^)	0.05	1.051	1.051 (0.941–1.181)	0.389
Bone mineral density (T score)	0.069	1.071	1.071 (0.571–1.895)	0.82
Dypertension history	− 0.23	0.795	0.795 (0.378–1.663)	0.541
Diabetes history	0.98	2.665	2.665 (0.731–17.18)	0.2
Time from fracture to surgery (4 weeks yes/no)	− 0.93	0.395	0.395 (0.185–0.842)	0.016
Posterior fascia oedema	− 0.989	0.372	0.372 (0.161–0.886)	0.022
IVC	− 0.988	0.372	0.372 (0.146–1.003)	0.042
Sarcopenia	− 1.353	0.259	0.259 (0.117–0.569)	0.001
ISLD	− 1.149	0.317	0.317 (0.145–0.699)	0.004
Bone cement distribution (Confluent/ Separated)	− 0.042	0.959	0.959 (0.379–2.769)	0.933
Cement volume (ml)	0.007	1.008	1.008 (0.807–1.27)	0.948
Bone cement leakage	− 0.424	0.654	0.654 (0.249–1.931)	0.409
Pre-LKA (°)	− 0.023	0.977	0.977 (0.932–1.027)	0.351
Pro-LKA (°)	− 0.021	0.979	0.979 (0.926–1.04)	0.481
Pre-AVH (%)	0.076	1.079	1.079 (0.993–1.172)	0.071
Pro-AVH (%)	0.015	1.015	1.015 (0.914–1.118)	0.772

### Multivariate analysis

A multivariate analysis was conducted using potential predictive features identified through LASSO regression analysis. This analysis revealed that posterior fascial oedema (OR 9.10; 95% CI 2.96–30.73; *P* = 0.00), IVC (OR 8.67; 95% CI 2.47–32.16; *P* = 0.001), time from fracture to surgery (OR 6.55; 95% CI 2.52–18.49; *P* = 0.00), sarcopenia (OR 9.73; 95% CI 3.53–29.67; *P* = 0.00), and ISLD (OR 5.06, 95% CI 1.92–14.02; *P* = 0.001) were considered as potential independent risk factors for residual LBP. These findings were summarized in Table 4.

**Table 4:** Results of multivariate analysis of variables associated with postoperative residual LBP in patients with OVCFs

Variables	β	OR	HR (95% CI)	*P* value
Posterior fascia oedema	2.208	9.1	9.1 (2.961–30.73)	0
IVC	2.16	8.67	8.67 (2.47–32.16)	0.001
Sarcopenia	2.276	9.734	9.734 (3.531–29.67)	0
ISLD	1.622	5.061	5.061 (1.918–14.02)	0.001
Time from fracture to surgery (4 weeks yes/no)	1.88	6.554	6.554 (2.521–18.49)	0

### Predictive model construction

**Fig. 8:**
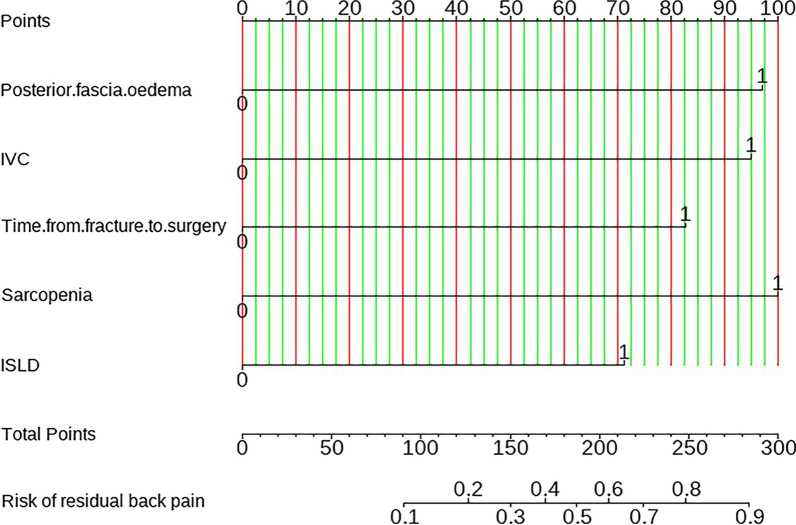
Predictive nomogram for residual LBP after percutaneous vertebroplasty (1-Yes, 0-No)

**Fig. 9:**
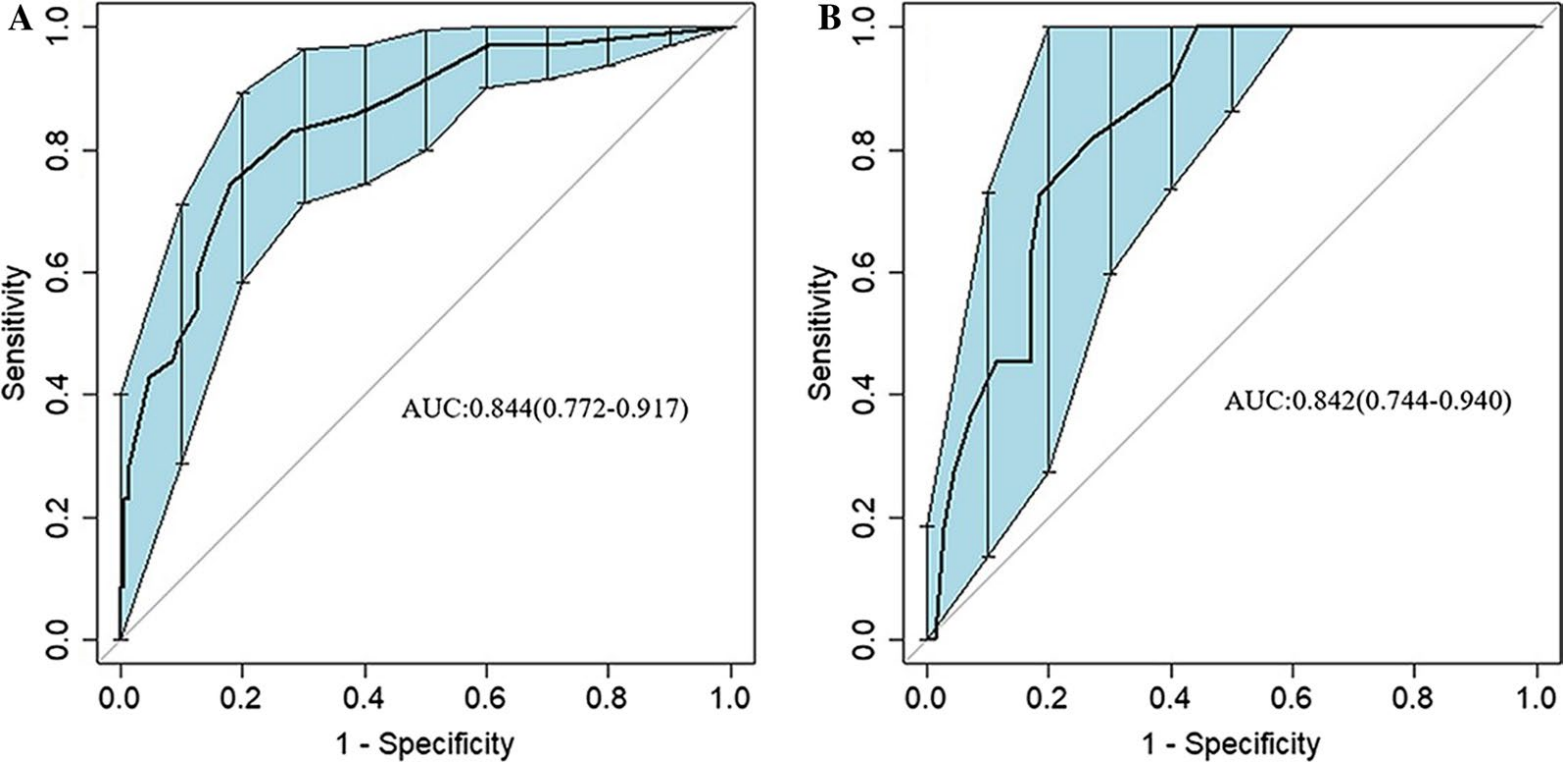
Receiver operating characteristic curve (ROC) validation of the residual LBP risk nomogram prediction. **A** ROC curve for the prediction model. **B** ROC curve of the internal validation

**Fig. 10:**
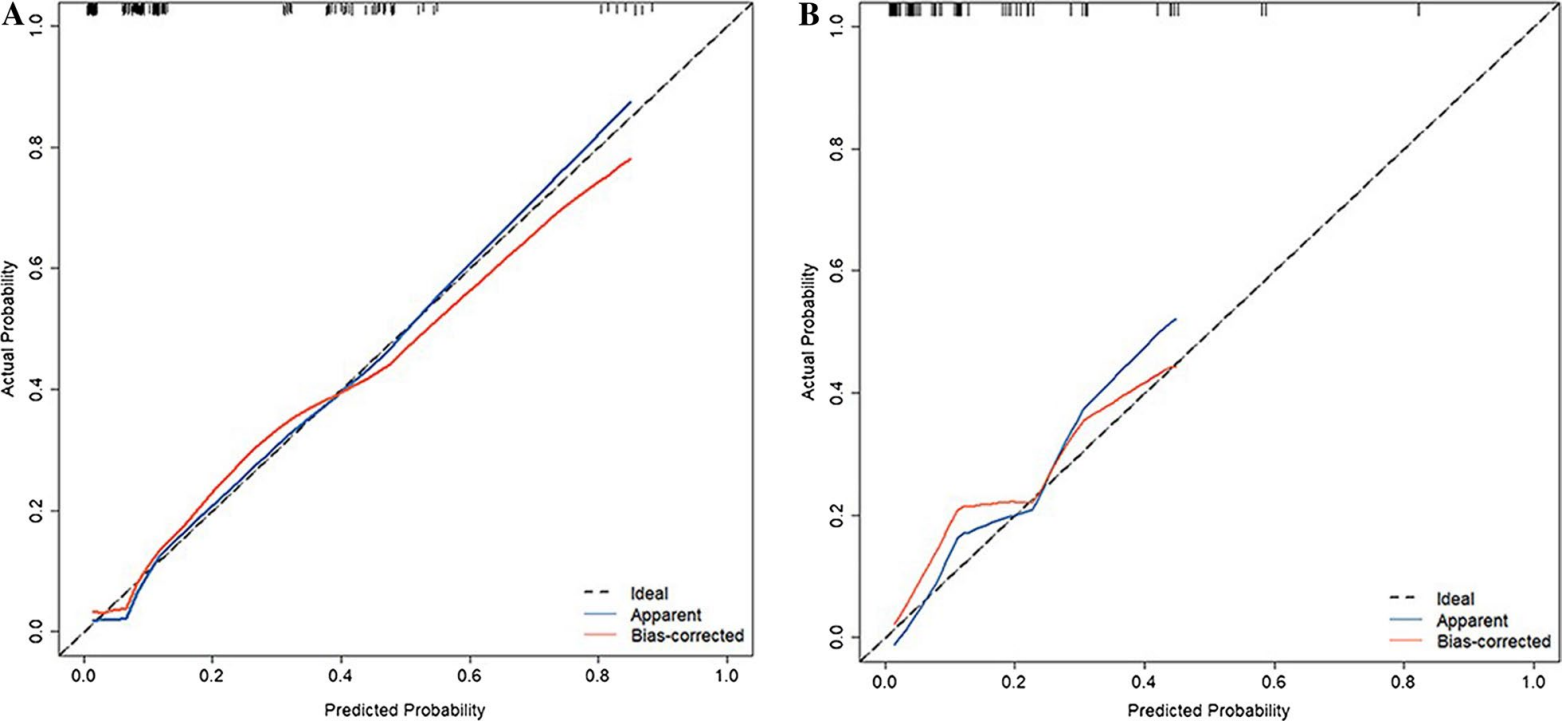
Calibration curves of the predictive residual LBP risk nomogram. **A** calibration curve for the prediction model. **B** calibration curve of the internal validation

**Fig. 11:**
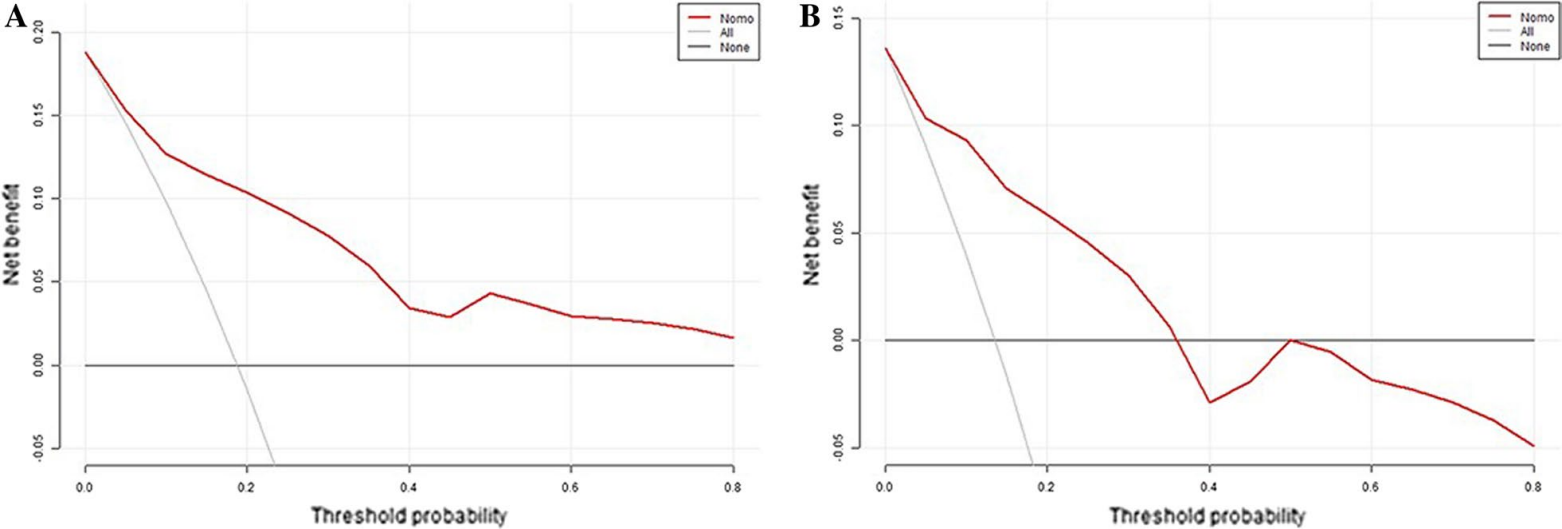
Decision curve analysis for the residual LBP risk nomogram.  **A** decision curve analysis of the predictive model. **B** decision curve analysis of the internal validation

**Fig. 12:**
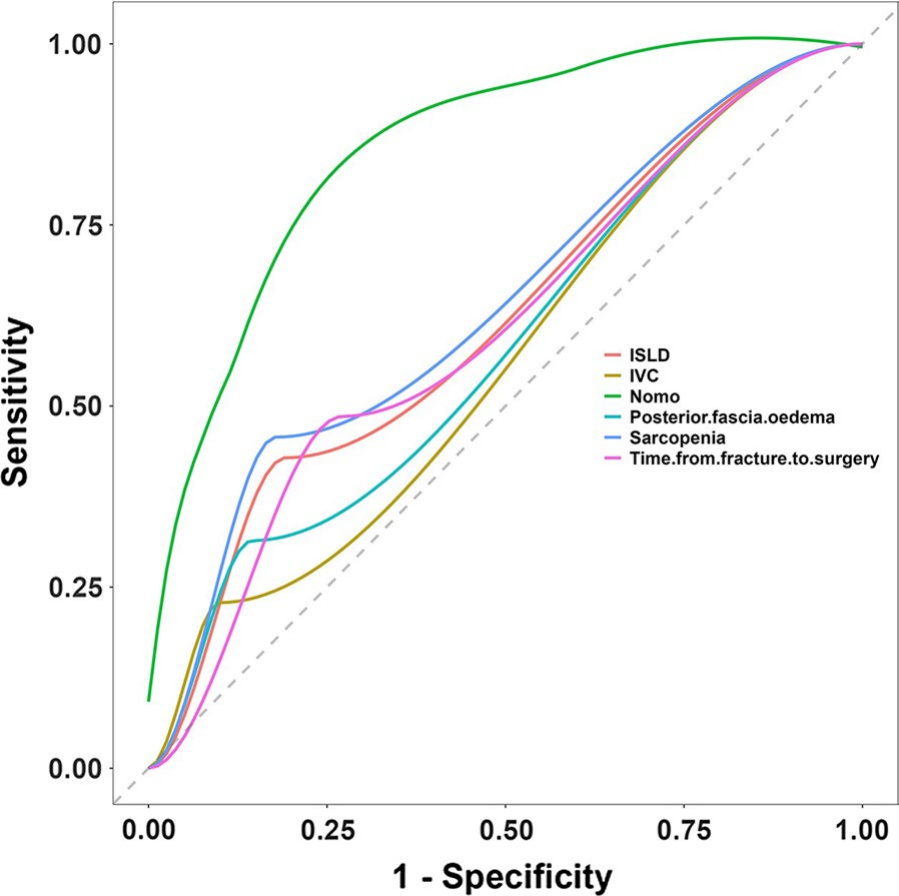
Rationality curve analysis for the residual LBP risk nomogram

Following the identification of predictors through multivariate analysis, we constructed a nomogram (Fig. 8). Additionally, Receiver Operating Characteristic (ROC) curves were plotted. In the training set, the model's discrimination, measured by the AUC, was 0.844 (95% CI 0.772–0.917), while in the validation set, it was 0.842 (95% CI 0.744–0.940). This indicates that the model exhibited strong predictive accuracy and discrimination. (Fig. 9). To ensure the predictive model's reliability, we performed calibration using calibration plots and Hosmer–Lemeshow tests. The calibration curves exhibited an excellent fit between the predictive model and the validation set. Furthermore, the Hosmer–Lemeshow test demonstrated a high level of agreement between predicted and actual probabilities (training set, *P* = 0.841; validation set, *P* = 0.345) (Fig. 10). The Decision Curve Analysis (DCA) results for both the training and validation sets demonstrated a significant net clinical benefit of the numerical model (Fig. 11). For a more comprehensive assessment, we compared the ROC curve of the prediction nomogram with that of the model utilizing single predictors, as depicted in Fig. 12. Remarkably, the AUCs of the single predictors were consistently smaller than those of the predictive model, underscoring the model's robust performance.

## Discussion

PKP is a commonly used and effective treatment in clinical practice, surpassing conservative methods in significantly reducing long-term mortality. However, PKP treatment carries certain complications [ [17]], including postoperative adjacent vertebral fractures, pulmonary embolism, cement leakage, and postoperative residual LBP. Among these, the persistence of LBP post-surgery substantially diminishes patients' satisfaction and quality of life. The specific causes of this residual LBP after PKP treatment remain unclear. Our clinical observations have revealed a multitude of factors influencing postoperative LBP. In this study, we delved into the examination of factors impacting postoperative residual LBP, specifically in a total of 267 patients with thoracolumbar OVCFs. Through the development of a predictive model for residual LBP following PKP treatment in patients with thoracolumbar OVCFs, we identified five potential independent risk factors: posterior fascial oedema, IVC, time from fracture to surgery, sarcopenia, and degeneration of the interspinous ligament. Significantly, the ROC analysis demonstrated an AUC of 0.844 (95% CI 0.772–0.917), signifying the model's robust discrimination and calibration. Moreover, the DCA results in both the training and validation sets affirmed the model's substantial net clinical benefit. Moreover, a plausibility analysis of the predictive model again showed that the model was reasonable.

IVC is a condition typically associated with older individuals who have severe osteoporosis, and it is widely believed to be attributed to ischemic necrosis. Furthermore, as noted by Kim [18], the IVC sign was predominantly located at the thoracolumbar junction (81%). Considering the heightened activity and increased mechanical stress experienced by this region, biomechanics was also believed to have a notable impact on the formation of the IVC sign. Therefore, IVC is considered indicative of spinal instability. In the treatment of OVCFs accompanied by IVC, PVP or PKP is often the primary choice [19]. However, several studies have uncovered a strong association between IVC and long-term postoperative issues, such as residual low back pain, vertebral body collapse, and instability [20]. Li et al. [21] demonstrated that patients with OVCFs and IVC typically experience substantially higher VAS scores than those without IVCs. This could be attributed to the cement primarily filling the IVC, potentially leaving the rest of the vertebral body inadequately supported and treated. In alignment with these findings, our results also indicated that the preoperative presence of IVC was a significant risk factor for postoperative residual LBP following PKP.

Furthermore, despite posterior fascial oedema being categorized as a soft tissue injury, it remains a significant consideration in clinical practice. The majority of patients with OVCFs are caused by low-energy injury such as falls. Nevertheless, our study revealed that the presence of posterior fascial oedema in OVCFs patients is not uncommon (19.2%), consistent with previous research findings. Yang [22] and colleagues demonstrated that PKP effectively alleviated LBP in OVCFs patients, but the same relief was not observed in patients with posterior fascial oedema. Their conclusion pointed to a strong connection between posterior fascial oedema and residual LBP. The posterior spinal nerve roots' branches traverse through the thoracolumbar fascia (TLF). When fascial injury occurs, nerve compression, inflammatory factor stimulation, and soft tissue oedema emerge as potential culprits behind the associated pain. Additionally, research on models related to the induction of posterior fascial oedema had consistently shown that reducing oedema and inflammatory factors tended to correlate with gradual pain reduction [23]. Consistent with prior studies, multifactorial analysis underscores the status of posterior fascial oedema as an independent risk factor for post-PKP residual LBP.

Sarcopenia, an age-related condition, is both progressive and widespread [24]. It involves a reduction in muscle mass and function and is often closely linked to osteoporosis. Multiple studies have indicated that nutritional disorders can be the root cause of both sarcopenia and osteoporosis, leading to functional impairment. It is not uncommon for patients to grapple with both conditions [25]. Muscles play a pivotal role in maintaining spinal stability. Sho [26] and colleagues have illustrated how sarcopenia impacts LBP and daily life for osteoporosis patients. Additionally, Yu [27] and team have demonstrated that sarcopenia diminishes the clinical efficacy of PKP. Hence, it is reasonable to hypothesize that sarcopenia may influence the alleviation of LBP following PKP in patients with OVCFs. In this study, we assessed muscle mass using lumbar spine CT. The measurement of the total psoas major muscle area (TPA) at the level of the third lumbar vertebra's transverse process offers a simple and rapid method to evaluate sarcopenia [28, 29]. Our findings indicated a notably higher occurrence of sarcopenia in the residual LBP group when compared to the control group. Multifactorial regression analysis further confirmed that sarcopenia stands as an independent risk factor for post-PKP residual LBP. Consequently, for OVCFs patients with sarcopenia, the focus should extend beyond merely enhancing vertebral stiffness to also encompass muscle mass improvement. In clinical practice, the treatment of sarcopenia recommends a combination of exercise and multiple nutritional supplements, including protein and vitamin D supplementation [30, 31].

We have observed that some patients with OVCFs continue to experience persistent LBP even after a period of conservative treatment, significantly impacting their daily lives. In clinical practice, surgical intervention is often recommended. While numerous previous studies have compared the effectiveness of conservative treatment and vertebral augmentation, little attention has been given to the timing of vertebral augmentation. Akihito [32] and colleagues discovered that early surgical treatment in OVCFs patients reduced the incidence of postoperative LBP and re-fractures. However, for those OVCFs patients who still experience residual LBP after surgery, some studies have concluded that the timing of vertebral augmentation is not an independent risk factor [33]. In our study, we utilized a 4-week period from injury to surgery as a reference point and found that the proportion of patients with an injury-to-surgery interval exceeding 4 weeks was higher in the group with residual LBP compared to the control group. This led us to propose that the timing of vertebral augmentation may indeed be a significant factor influencing the development of residual LBP following surgery in OVCFs patients (OR 6.554, *P* = 0.00). This could be attributed to various factors: (1) It might be associated with the delayed formation of osseous junctions or pseudoarthrosis, often stemming from the delayed treatment of compression fractures. Such delays are known to contribute to chronic pain that ensues after an injury [34–36]. (2) The formation of primitive callus typically takes place around four weeks post-fracture and undergoing vertebral kyphoplasty at a later date (more than 4 weeks) is not conducive to alignment improvement.

Degenerative changes in the lumbar spine rank among the primary causes of LBP and disability in the elderly [37]. Typically, we place our focus on alterations in intervertebral disks while overlooking the significance of non-disk structures within the spine, including the facet joints, paraspinal muscles, and spinal ligaments. Among these structures, the interspinous ligament holds a pivotal position within the posterior spinal ligament complex. It plays a crucial role in spinal flexion. When the body undergoes excessive forward bending or experiences an energetic impact, the posterior column structures of the lumbar spine come under increased tension and load, with the interspinous ligament often bearing the brunt of the injury. Multiple studies have assessed the interspinous ligament's role through biomechanical tests [38] and through the observation of anatomical, biochemical, and pathological changes in degenerative spines. Maes [39] and colleagues have concluded that the degeneration of posterior column ligaments is a significant contributor to LBP. In the investigation of changes within the interspinous ligament, MRI has established itself as the primary diagnostic method of choice. Keorochana et al. categorized ISLD into four types [40, 41]. However, our study encompassed cases beyond mild degeneration. In line with prior research, multifactorial analysis demonstrated that ISLD stands as a significant risk factor for persistent LBP following PKP (OR 5.061, *P* = 0.001). Consequently, we created a nomogram through the analysis of potential risk factors to predict the occurrence of residual LBP. This model proves to be a viable tool for risk assessment, enabling early identification of OVCFs patients with a high risk of residual LBP. With this knowledge, individualized strategies can be crafted for these patients during the perioperative period.

Our study has several limitations. First, it is important to acknowledge that our study is retrospective and has a restricted sample size. Second, our postoperative follow-up period is relatively short, and we acknowledge the importance of conducting studies with extended follow-up durations and multiple assessment time points. Third, it is essential to highlight that this study is conducted at a single center, and the validation of our findings would benefit from future multicenter studies conducted in different hospitals.

## Conclusion

We have created a novel numerical model that can precisely forecast the potential risk factors associated with the onset of LBP following PKP in patients with thoracolumbar OVCFs. This model is poised to aid in the early identification of OVCFs patients at a heightened risk of enduring residual LBP, offering valuable insights to guide clinical decision-making.

## Data Availability

The authors will provide the raw data supporting the conclusions of this paper without reservation.

## Abbreviations

OVCFs: Osteoporotic vertebral compression fractures
PKP: Percutaneous kyphoplasty
LBP: Low back pain
ROC: Receiver operating characteristic
DCA: Decision curve analysis
TLFI: Thoracolumbar fascia injury
IVC: Intravertebral vacuum cleft
ISLD: Interspinous ligament degeneration
BTMs: Biochemical markers of bone turnover
bALP: Bone alkaline phosphatase
PINP: Procollagen type I N propeptide
ODI: Oswestry disability index
VAS: Visual analogue scale
AVH: Anterior vertebral height
LKA: Local kyphotic angle
MRI: Magnetic resonance imaging
TPA: Total psoas area
